# Performance and risk factors associated with first antibiotic treatment in two herds, raising pigs without antibiotics

**DOI:** 10.1186/s40813-021-00198-y

**Published:** 2021-02-17

**Authors:** J. C. Lynegaard, I. Larsen, C. F. Hansen, J. P. Nielsen, C. Amdi

**Affiliations:** 1 Department of Veterinary and Animal Sciences, Faculty of Health and Medical Sciences, University of Copenhagen, Grønnegårdsvej 2, DK-1870 Frederiksberg C, Copenhagen, Denmark; 2 Danish Pig Research Centre SEGES, Danish Agriculture and Food Council, Axeltorv 3, DK-1609 Copenhagen V, Denmark

**Keywords:** Antibiotics, Bodyweight, Diarrhoea, Gender, Growth, Pigs

## Abstract

**Background:**

Antibiotic (AB) consumption in production animals has a high awareness among politicians and consumers due to the risk of selection for AB resistance among potentially zoonotic bacteria. However, AB treatment of animals is at times necessary to treat diseases and ensure the wellbeing of the animals we take into our care. Raised without antibiotics (RWA) is a concept where pigs are individually ear-tagged for tracking, and if pigs are AB treated, they lose their RWA status. At slaughter, the farmer receives an additional price for non-AB treated pigs. The objective of this study was to identify risk factors for AB treatment and to investigate growth performance of pigs in two Danish RWA herds.

**Results:**

A total of 518 pigs in herd A and 436 pigs in herd B, were individually ear-tagged and subjected to weekly investigations of AB treatment status from birth to 12 weeks of age. Bodyweight was recorded at birth, 2, 4 and 12 weeks of age. The results showed, that at 12 weeks of age, 82 of 518 liveborn pigs were AB treated in herd A and 31 of 436 liveborn pigs were AB treated in herd B. Individual pigs that required AB treatment had a reduced average daily gain from day 0 to 28 in both herds (herd A, *P* <  0.001; herd B, *P* = 0.062) and from day 0 to 84 in herd A (*P* <  0.001). Additionally, significant risk factors for AB treatment were identified as a low bodyweight in herd A, whereas barrows and litters with less than 19 piglets were the main risk factors in herd B.

**Conclusion:**

The results suggests that in order to reduce AB treatments particular attention should be addressed to smaller pigs and barrows in RWA herds. In these two Danish RWA herds from this study it was possible for 64 and 68% pigs to reach 12 weeks of life without any AB treatments.

**Supplementary Information:**

The online version contains supplementary material available at 10.1186/s40813-021-00198-y.

## Background

The use of antibiotics (AB) in production animals is a debated subject, but from an animal welfare point of view, treating sick pigs suffering from a bacterial infection with AB is necessary. Antimicrobial resistance has been the main driver of the consumer interest for livestock raised without antibiotics (RWA) [1–3].

In Denmark, the production concept RWA began with six Danish pig producers in collaboration with the meat industry (Danish Crown, Randers, Denmark) in 2015, which has increased to more than 50 producers rearing RWA pigs in 2018 [3]. The RWA concept is based on preventive measures, including increased focus on hygiene, vaccines and feeding programmes, as well as improved management, as alternatives to AB treatments [4]. Individual AB treatment for welfare reasons is allowed in RWA herds, but pigs receiving AB treatment will lose their RWA status. In practice this is handled by selecting and ear tagging those pigs enrolled in the RWA programme before 4 days of age. It is up to the pig producer to decide which pigs should be included and subsequently ear tagged for RWA production. If an ear-tagged pig needs AB treatment the ear-tag is removed, and the pig will lose its RWA status and the RWA premium at slaughter.

It is well known that pigs with a low birthweight (BiW) or low weaning weight have a reduced average daily gain (ADG) throughout their lifespan [5–7]. However, there is limited scientific information available regarding growth performance in RWA productions and factors influencing AB treatments of individual pigs in RWA piggeries.

Therefore, the objectives of this study were to (1) determine when pigs in two Danish RWA herds receive AB treatment from birth to 12 weeks of age, (2) to identify risk factors for AB treatment and (3) to describe the growth performance of AB treated pigs. We hypothesized that low BiW piglets were at an increased risk of AB treatment and that AB treatment had an effect on growth performance.

## Methods

### Study design and study population

A quantitative observational cohort study was performed in two Danish RWA herds (chosen by convenient sampling) to investigate their performance and risk factors associated with AB treatments. The participating herds were part of the Danish programme of raising pigs without AB in collaboration with Danish Crown (Randers, Denmark). Both farms operated as farrow-to-finish systems with Danish Landrace x Yorkshire sows mated with Duroc semen (Danbred, Denmark), and the production included 985 sows in herd A and 400 sows herd B.

The study period included one batch of pigs in both herds and the pigs were included in the study from birth to approximately 12 weeks of age (~ 30 kg bodyweight (BW)). The study ended at 12 weeks just before pigs were moved to a finishing site, and because fewer AB treatments were expected in the finishing stages [3]. All piglets born alive during three consecutive days (518 piglets in herd A and 436 piglets in herd B) were ear-tagged within 12 h of birth with unique id-numbers. Piglets further received a RWA ear-tag by the herd staff in the opposite ear within the first 4 days postpartum. If a pig required AB treatment due to disease or injury the RWA ear-tag was removed but the unique id-number kept, and the pig would remain in the herd but was sold as a conventional (non-RWA) slaughter pig.

### Housing and management

In the farrowing unit, sows were housed in farrowing crates with a heating lamp over the creep area for the piglets.

#### Herd A

Litters were equalized at farrowing by cross-fostering piglets and creating two-step nursing sows (three-week old pigs are weaned from one sow and this sow receives a one-week old litter to nurse until weaning at 4 weeks, and the sow that gave her one-week old litter receives new surplus piglets) [8], were both gilts and sows received approximately 16 piglets. At three to 4 days of age, male pigs were castrated following analgesia (Metacam, Boehringer Ingelheim Animal Health Nordic A/S, Copenhagen, Denmark), all piglets where tail-docked, given iron by injection (Uniferon, Salfarm A/S, Kolding, Denmark) and orally treated against coccidiosis (Toltarox, Dechra Veterinary Products A/S, Uldum, Denmark). Furthermore, piglets that had not received AB treatment and clearly marked with spray, received an RWA ear-tag. In this herd, ten pigs were chosen for own gilt production and did not receive a RWA ear-tag, these pigs were therefore excluded from the study (never RWA-tagged).

Piglets were weaned between three to five weeks of age, when the employees estimated that they had reached a desired BW of around 5 to 6 kg. Some of the bigger piglets were moved individually to make more room, and the remaining litter were weaned as one. In the nursery unit pigs were randomly divided into pens and only moved during the nursery period if they required special attention in a sick pen. Pigs stayed in the nursery unit for about 8 weeks. The nursery unit contained two sections, with 18 pens in each section with a holding capacity of about 30–40 pigs. Each pen had about 30% slatted floors and a covered area over about 40% of the solid floor. The pens were further equipped with a water nipple and a feeding trough with ad libitum access to feed.

Pigs were vaccinated orally with Coliprotec F4/F18 (Elanco, Herlev, Denmark) prior to weaning, vaccinated orally against *Lawsonia intracellularis* 4 days post-weaning with Enterisol Ileitis Vet (Boehringer Ingelheim Animal health Nordic A/S, Copenhagen, Denmark), and intramuscularly against PCV2 and *Mycoplasma hypneumoniae* with Porcilis PCV M Hyo (MSD Animal Health, Copenhagen, Denmark) as well as Swine Influenza Virus with Respiporc FLU3 (CEVA Animal Health, Vejle, Denmark) one week after weaning.

#### Herd B

During the first 12 h after birth, all litters were split-suckled to increase colostrum supply for small piglets. Litters were equalized according to piglet size and both gilts and sows received 15 to 16 piglets, but gilts only received piglets from multi-parous sows and entire gilt litters were therefore moved to older sows.

Four to five days post-farrowing, male pigs were castrated, and all piglets were tail docked following analgesia (Procamidor Comp Vet, Salfarm A/S, Kolding, Denmark), allocated an iron injection (Solofer, Vitfoss, Denmark) and an oral anticoccidial treatment (Espacox, Biovet, Denmark). Furthermore, piglets that had not received AB treatment and clearly marked with spray, received a RWA ear-tag. A strategy for this herd was to leave out RWA ear tagging of small (approximately < 800 g) and unthrifty piglets at birth.

Pigs were weaned between three to five weeks of age, when the employees estimated that they had reached a desired BW of around 5 to 6 kg, and moved to a weaner unit, containing eight sections with a varying number of pens. Some big piglets were moved individually to make more room, and the remaining litter were weaned as one. The newly weaned pigs were divided between pens according to size, with a holding capacity of 25 to 30 pigs per pen and were only moved during the weaner period if they required special attention in a sick pen. They were housed in the weaner unit for around seven weeks (BW 25 to 27 kg). The herd had four different sections for weaners with a varying number of pens of different sizes. Each pen had approximately 30% slatted floors and a cover over about 40% of the solid floor. Furthermore, each pen had a drinking nibble and a feeding trough with ad libitum access to feed.

The newly weaned pigs were vaccinated intradermally with Porcilis PCV ID (MSD Animal Health, Copenhagen, Denmark) against PCV2, and orally with Coliprotec F4/F18 (Elanco, Herlev, Denmark) against *E. coli* infection.

In both herds, all AB treatments were carried out by the herd staff after instructions by the practising veterinarian and according with Danish legislation. Treatments were administered when recognizing specific signs of disease, as described by the veterinarian. Treatments were discussed and checked in both herds and concluded to be in order with instructions.

### Feeding

#### Herd A

Sows had ad libitum access to drinking water and were fed a meal diet three times daily, according with Danish recommendations [9]. The feed was mixed on farm, based on a mix of barley and wheat, de-hulled soybean meal, sugar beet pulp and a premix (Vilomix A/S, Mørke, Denmark). From day 10, all piglets received post-weaning dry feed on the floor in the creep area.

While in the nursery, pigs had ad libitum access to drinking water and were fed a three-phase diet according with Danish recommendations [9]. Phase 1 were fed from weaning to approximately 9 kg, phase 2 was offered from 9 kg to approximately 15 kg, and phase 3 from 15 kg to approximately 30 kg. The diets were mixed on farm, based on barley, wheat, de-hulled soybean meal and a premix (Vilomix A/S, Mørke, Denmark). Diet formulations and chemical compositions can be seen in Additional file 1. The phase 1 diet contained 1500 ppm zinc oxide. Additionally, the weaners were given 0.2% organic acids in the water for approximately 4 weeks after weaning, containing formic and lactic acids (MS Goldfeed Prestige, MS Schippers, The Netherlands).

#### Herd B

Sows had ad libitum access to drinking water and were fed a commercially formulated diet three times daily (Hornsyld Købmandsgaard A/S, Hornsyld, Denmark), according with Danish recommendations [9]. The diet was based on wheat, barley and sunflower meal and mixed on farm. From day 2 to 14, milk replacer (Danmilk Supreme 1.0, Agilia, Videbaek, Denmark) was offered in pens were sows nursed the smallest piglets. From around day 10, all piglets received post-weaning dry feed on the floor of the farrowing pen (A-One Combat 4, Skive, Denmark).

While in the weaner unit, pigs had ad libitum access to drinking water and ad libitum access to two to three commercially formulated dry-feed diets, based on Danish recommendations [9] and mixed on farm. The first diet were only offered to the smallest pigs from weaning until they reached a BW of around 7 kg (A-One Combat 4, Skive, Denmark), the second diet were allocated to the remaining pigs from weaning to around 10 kg, as well as the smallest pigs from 7 to 10 kg (Vilofoss, Fredericia, Denmark), and the last diet were allocated to all pigs from 10 kg and until they were moved at around 25 kg (Vilofoss, Fredericia, Denmark). Diet formulations and chemical composition can be seen in Additional file 2. The weaner diets did not include any medicinal zinc oxide. All weaners were allocated an acid mixture through the drinking water of medicinal mixer containing 0.2% formic acid and propionic acid (Acid One, Brenntag Nordic A/S, Ballerup, Denmark). If the employees observed any diarrhoea outbreaks in the weaner unit, the pigs were allocated potato flour containing medicinal zinc (2500 ppm) on the pen floor (A-one Denmark, Skive, Denmark).

### Recordings

At the day of farrowing, recordings of litters included; id of sow, parity and the number of live born and stillborn piglets. Recordings of individual piglets included; gender and whether the piglets were subjected to intra-uterine growth restriction (IUGR) based on Chevaux [10] and Hales et al. [11]. Individual BW of the pigs were recorded on a scale (Bjerringbro Vægte ApS, Bjerringbro, Denmark) at the day of farrowing, at two, four and 12 weeks of age (Day 0, 12, 24 and 82 for herd A, and day 0, 12, 26 and 82 for herd B).

All pigs were examined at 2, 4, 5, 6, 7, 8, and 12 weeks of age in herd A, and at 1, 2, 3, 4, 5, 6, 7, 8 10 and 12 weeks of age in herd B, in order to check whether the RWA ear-tag was present. Loss of RWA ear-tag was interpreted as an AB treatment.

While the piglets were in the farrowing unit, id of the sows and the number of piglets by each sow were recorded every week. When pigs died or were euthanized during the study, it was noted, and pigs were stored in a freezer until further examination (data not shown). Furthermore, pen number was recorded each week in the weaner unit, and the number of moves between both sows and pens were registered.

### Statistical analysis

All calculations and statistical analysis were performed using the statistic program R (vers. 3.4.0) with pig as the experimental unit. Significance was accepted at *P* <  0.05 and *P* <  0.10 was defined as a trend.

Average daily gain was calculated based on BW measurements at day 0, 12, 24 in herd A vs. 26 in herd B and day 82 in both herds. Growth performance was analyzed using the linear mixed-effect models in R, including BiW as a covariate, RWA-status as a factor and sow as a random effect. Gender and weaning age were excluded from the growth model, as they were not significant. Interactions were further tested and deemed not significant. The model assumption of normality and homogeneity were checked with residual plots.

Risk factor analysis for AB treatment were performed at three different time points (at 2, 4 and 12 weeks of age) using the generalized mixed linear model in R (logistic regression). The RWA-status was included in the model as a fixed effect. All numeric variables were divided into categorical factors, based on mean and two standard deviations from the mean (Table 1). Risk factors were tested in a logistic regression model for individual significance, and factors with a *P* <  0.10 were included in a multivariable model. Risk factors on both sow and pig level were analysed in the same multivariable model and backwards testing was performed for exclusion of factors with a significance level above *P* <  0.05. The results are presented as odds ratios (OR) and confidence intervals.

**Table 1:** Number of untreated (−) and antibiotics (AB) treated (+) pigs per risk factor, presented at 2, 4 and 12 weeks of age. Missing, dead and pigs never receiving a raised without antibiotics (RWA) ear-tag are excluded from this table

	Herd A									Herd B								
	2 weeks			4 weeks			12 weeks			2 weeks			4 weeks			12 weeks		
AB treated	All	–	+	All	–	+	All	–	+	All	–	+	All	–	+	All	–	+
Gender	NS			NS			NS			**			**			NS		
Female	233	207	26	234	191	43	212	178	34	170	167	3	168	163	5	158	146	12
Barrow	230	207	23	223	180	43	203	155	48	182	167	15	181	163	18	171	152	19
IUGR^a^ at birth	NS			NS			NS			NS			NS			NS		
Yes	26	22	4	27	21	6	23	20	3	18	17	1	18	17	1	16	15	1
No	437	392	45	430	350	80	392	313	79	334	317	17	331	309	22	313	283	30
BiW^a^	NS			*			NS			NS			NS			NS		
< 1 kg	95	83	12	93	68	25	79	59	20	38	35	3	38	34	4	36	32	4
1–1.6 kg	296	267	29	290	241	49	264	214	50	241	232	9	239	227	12	225	206	19
> 1.6 kg	63	56	7	62	54	8	62	53	9	66	60	6	65	58	7	61	53	8
BW^a^ 2 weeks	**			**			**			NS			NS			NS		
< 2.7 kg	54	39	15	50	29	21	38	25	13	69	65	4	68	62	6	65	57	8
2.7–4.5 kg	314	278	36	300	249	51	277	221	56	207	196	11	206	192	14	190	172	18
> 4.5 kg	97	92	5	96	87	9	92	82	9	55	53	2	54	53	1	53	51	2
BW 4 weeks				**			**						NS			NS		
< 4.5 kg	–	–	–	62	31	31	55	22	33	–	–	–	24	21	3	22	17	5
4.5–7.2 kg	–	–	–	265	225	40	241	202	39	–	–	–	270	253	17	255	234	21
> 7.2 kg	–	–	–	82	77	5	79	74	5	–	–	–	39	38	1	37	34	3
BW 12 weeks							**									NS		
< 23 kg	–	–	–	–	–	–	54	33	21	–	–	–		–	–	58	50	8
23–36 kg	–	–	–	–	–	–	248	202	46	–	–	–		–	–	227	207	20
> 36 kg	–	–	–	–	–	–	104	93	11	–	–	–		–	–	38	35	3
Sow parity	NS			NS			NS			*			*			NS		
1	50	43	7	48	38	10	44	34	10	41	35	6	40	34	6	37	30	7
> 1	413	371	42	406	332	74	368	298	70	311	299	12	309	292	17	292	268	24
Total born piglets (sow)	NS			NS			NS			**			*			*		
< 19	70	62	8	69	57	12	65	52	13	22	17	5	21	16	5	20	15	5
19–25	232	213	19	229	194	35	211	177	34	234	226	8	233	222	11	224	210	14
> 25	99	83	16	97	71	26	83	62	21	81	78	3	80	76	4	71	62	9
Transfers between sows				NS									NS					
0	–	–	–	184	151	33	–	–	–	–	–	–	21	20	1	–	–	–
1	–	–	–	265	214	51	–	–	–	–	–	–	101	92	9	–	–	–
2	–	–	–	8	6	2	–	–	–	–	–	–	175	163	12	–	–	–
3	–	–	–	–	–	–	–	–	–	–	–	–	44	44	0	–	–	–
4	–	–	–	–	–	–	–	–	–	–	–	–	8	7	1	–	–	–
Transfers in weaner unit							*									NS		
0	–	–	–	–	–	–	246	208	38	–	–	–	–	–	–	92	83	9
1	–	–	–	–	–	–	90	71	19	–	–	–	–	–	–	66	58	8
2	–	–	–	–	–	–	67	44	23	–	–	–	–	–	–	130	120	10
3	–	–	–	–	–	–	12	10	2	–	–	–	–	–	–	36	32	4
4	–	–	–	–	–	–		–	–	–	–	–	–	–	–	5	5	0

^a^*IUGR* intra-uterine growth restricted, *BiW* birthweight, *BW* bodyweight

*NS* non-significant, * *P* < 0.05, ** *P* < 0.01. Logistic model and *P*-values association tested by Chi square-test

### Missing pigs

In herd A 35 pigs and in herd B 23 pigs were not present at the end of the trial nor were they recorded as dead by the herd personnel. As many pigs went missing throughout the experiment, they cannot all be assumed deceased without being recorded, but we expect some have lost their ear-tag and others were overlooked in the pens.

## Results

### Descriptive results

The study included 518 piglets from herd A with an average BiW of 1.24 (± 0.32) kg. In herd B, 436 piglets were ear-tagged at birth with an average BiW of 1.27 (± 0.32) kg, however, 54 piglets never received an RWA ear-tag due to the herd strategy of leaving out small and unthrifty piglets. Only pigs receiving an RWA ear-tag at birth were included in the risk factor analyses. The number of untreated and AB treated pigs on a weekly basis from birth to 12 weeks of age are displayed in Table 2. Individual reason for AB treatment were recorded in herd B during the first 2 weeks of the trial. During this time, 14 piglets were treated for diarrhoea and 23 piglets for leg injuries.

**Table 2:** The number of pigs as untreated, antibiotics (AB) treated, dead and missing from day 1 to 12 weeks of age in two Danish Raised without antibiotics (RWA) herds

	Herd A					Herd B				
Weeks of age	Untreated	AB treated	Dead (untreated/treated)	Missing pigs	Never RWA ear-tagged	Untreated	AB treated	Dead (untreated/treated)	Missing pigs	Never RWA ear-tagged
0	518	–	–	–	–	436	–	–	–	–
1	–	–	–	–	–	343	11	26 (1/25)	3	53
2	414	49	39 (9/30)	6	10	334	18	30 (3/25)	3	51
3	–	–	–	–	–	327	22	31 (4/25)	7	49
4	371	86	42 (10/32)	9	10	326	23	31 (4/25)	8	48
5	359	93	42 (10/32)	14	10	323	25	31 (4/25)	9	48
6	353	96	43 (11/32)	16	10	320	27	32 (5/25)	9	48
7	353	88	50 (15/35)	17	10	316	27	33 (6/25)	12	48
8	343	91	55 (17/38)	19	10	315	27	33 (6/25)	13	48
10	–	–	–	–	–	307	29	36 (8/26)	17	47
12	333	82	57 (18/39)	36	10	298	31	37 (9/26)	23	46
% at 12 weeks	64	16	11	7	2	68	7	8	5	11

In herd A, 161 pigs were weaned at 3 weeks of age and 268 pigs at 4 weeks of age. Whereas, in herd B, 183 pigs were weaned at 3 weeks, 207 pigs at 4 weeks and 45 pig at 5 weeks of age.

The effect of AB treatment on growth performance during the overall study period is summarized in Table 3. In herd A, AB treated pigs had a lower BW at 2, 4 and 12 weeks of age compared to the untreated pigs (*P* < 0.001). Moreover, AB treated pigs had a reduced ADG from birth to 4 weeks of age (untreated, ADG = 204 g/d; AB treated, ADG = 153 g/d, *P* < 0.001) as well as during the overall trial period from birth to 12 weeks of age (untreated, ADG = 368 g/d; AB treated, ADG = 313 g/d, *P* < 0.001).

**Table 3:** Growth performance of untreated and antibiotics (AB) treated pigs in two Danish “raised without antibiotics” (RWA) herds

	Herd A					Herd B				
	Untreated	AB treated	Never- RWA	SEM^2^	*P*-Value	Untreated	AB treated	Never-RWA	SEM^2^	*P*-value
BW^2^ (kg)										
Week 2	3.81^a^	3.20^bA^	3.63^bB^	0.087	< 0.001	3.44^a^	3.22^a^	3.39^a^	0.067	0.376
Week 4	6.13^a^	5.01^bA^	5.89^bB^	0.132	< 0.001	5.96^A^	5.48^B^	5.91^AB^	0.067	0.088
Week 12	31.3^a^	27.7^bA^	33.6^bB^	0.682	< 0.001	28.7^a^	26.8^a^	27.7^a^	0.625	0.154
ADG^2^ (g/day)										
Day 0–12	212^a^	158^b^	213^a^	3.76	< 0.001	168^a^	153^ab^	153^b^	4.94	< 0.001
Day 0–24/26^1^	204^a^	153^b^	202^a^	5.16	< 0.001	173^A^	155^B^	166^AB^	2.38	0.062
Day 24/26^1^–84	432^a^	374^b^	496^a^	10.7	< 0.001	403^a^	377^ab^	348^b^	11.1	0.001
Day 0–84	368^a^	313^b^	405^a^	8.13	< 0.001	330^a^	307^ab^	290^b^	7.7	< 0.001

^a,b^ Values within a row with different superscripts differ significantly at *P < 0.05* (values within a row with same superscript does not differ significantly)

^A,B^ Values within a row with different superscripts have a tendency to differ at *P < 0.10*

^1^Bodyweight were measured at day 24 in herd A, and day 26 in herd B

^2^*SEM* Standard error of mean, *BW* bodyweight, *ADG* average daily gain

In herd B, there was no significant difference between untreated and AB treated pigs at 2, 4 or 12 weeks of age (*P* > 0.05). However, there was a tendency for the AB treated pigs to have a lower BW at 2 weeks of age compared with the untreated pigs (untreated, BW = 5.96 kg; AB treated, BW = 5.48 kg, *P* = 0.088). Likewise, there was no significant difference in ADG between untreated and AB treated pigs in herd B, but only a tendency towards a lower ADG in AB treated pigs from birth to 4 weeks of age (untreated, ADG = 173 g/d; AB treated, ADG = 155 g/d, *P* = 0.062). On the other hand, pigs excluded from RWA production had a significantly lower ADG during the overall trial period compared to the untreated pigs (untreated, ADG = 330 g/d; never RWA ear tagged, ADG = 290 g/d, *P* < 0.001).

### Risk factor analysis

Included observational factors and the univariate association analysis are summarized in Table 1, with the factor levels and the number of pigs in each category presented for the two herds. The univariate analysis revealed that BiW, BW at 2, 4 and 12 weeks of age and transfers in the weaner unit were significant as a single factor in herd A (*P* < 0.05). Whereas number of total born piglets, parity and gender were significant as a single factor in herd B (*P* < 0.05). These factors were then included in the multivariable analysis for each herd and the results are presented in Table 4 for herd A and Table 5 for herd B.

**Table 4:** Multivariate risk factor analysis for antibiotics (AB) treated pigs in herd A

Factor	Level	Odds ratio	*P*-value^1^	CI^2^
Risk factors for AB treatment at two weeks of age				
BW^2^ at 2 weeks^3^	> 4.5 kg	Ref^2^		
	2.7–4.5 kg	1.72	< 0.001	0.66–5.20
	< 2.7 kg	7.08	< 0.001	2.55–23.00
Risk factors for AB treatment at four weeks of age				
BW at 4 weeks	> 7.2 kg	Ref		
	4.5–7.2 kg	2.33	0.395	0.61–8.92
	< 4.5 kg	8.09	0.044	1.39–46.07
Risk factors for AB treatment at 12 weeks of age				
BW at 4 weeks	> 7.2 kg	Ref		
	4.5–7.2 kg	2.84	0.083	1.18–8.49
	< 4.5 kg	23.54	< 0.001	8.89–75.30

^1^ Association tested by Chi square-test

^2^ *CI* Confidence intervals, *BW* bodyweight, *Ref* Reference when calculating odds ratio

^3^ For the number of pigs in each category, see Table 1

**Table 5:** Multivariate risk factor analysis for antibiotics (AB) treated pigs in herd B

Factor	Level	Odds ratio	*P*-value^1^	CI^2^
Risk factors for AB treatment at two weeks of age				
Gender^3^	Female	Ref^2^		
	Barrow	4.81	0.019	1.46–21.89
Litter size of mother sow	> 25	Ref^3^		
	19–25	0.89	0.983	0.25–5.14
	< 19	8.35	0.022	1.79–45.83
Risk factors for AB treatment at four weeks of age				
Gender	Female	Ref		
	Barrow	4.05	0.019	1.37–14.98
Litter size of mother sow	> 25	Ref^3^		
	19–25	0.97	0.999	0.30–3.72
	< 19	6.65	0.029	1.54–30.96
Risk factors for AB treatment at 12 weeks of age				
Litter size of mother sow	> 25	Ref		
	19–25	0.50	0.301	0.20–1.29
	< 19	2.29	0.029	0.63–7.72

^1^ Association tested by Chi square-test

^2^ CI = Confidence intervals; Ref = Reference when calculating odds ratio

^3^ For the number of pigs in each category, see Table 1

#### Herd A

Pigs with a BW below 2.7 kg had an increased risk of being AB treated at 2 weeks of age compared to pigs with a BW above 4.5 kg (< 2.7 kg, OR = 7.07, *P* < 0.001). Pigs with a BW below 4.5 kg at 4 weeks of age had a higher risk of being AB treatment at weaning compared to pigs with a BW above 7.2 kg (< 4.5 kg, OR = 8.09, *P* = 0.044). Lastly, pigs with a BW below 4.5 kg at 4 weeks of age had an increased risk of being AB treated at 12 weeks of age compared to pigs with a BW above 7.2 kg (< 4.5 kg, OR = 23.54, *P* < 0.001).

#### Herd B

Barrows had a higher risk of being AB treated at both 2 and 4 weeks of age compared with female pigs (2 weeks: barrow, OR = 4.81, *P* = 0.019; 4 weeks: barrow, 4.05, *P* = 0.019). Additionally, pigs from litters of < 19 live born had a significantly higher risk of being AB treated at both 2, 4 and 12 weeks of age compared to pigs from a litter above 25 liveborn (2 weeks: < 19, OR = 8.35, *P* < 0.001; 4 weeks: < 19, OR = 6.65, *P* = 0.019; 12 weeks: < 19, OR = 2.29, *P* = 0.029).

## Discussion

Danish RWA production aims at avoiding AB treatments throughout the production, however some pigs suffering from bacterial diseases require AB treatment for animal welfare reasons. In this cohort study based on results from two Danish RWA herds, we investigated the percentage of pigs that did not need AB treatment and the underlying risk factors for the AB treatments.

Sixty-four percentage of live born pigs in herd A and 68% of live born pigs in herd B were not AB treated before 12 weeks of age. The two study herds had very different treatment strategies, as herd B excluded 53 piglets from RWA production, whereas herd A only excluded a few pigs for breeding purposes. This was also reflected in the results as 19% of live born pigs in herd A received AB treatment before 12 weeks of age, compared to 7% of live born pigs in herd B. This suggest that pigs excluded from RWA production in herd B would probably have received an AB treatment before 12 weeks of age. At the same time, since 7–19% of pigs still receive AB treatment before 12 weeks of agein a RWA production, it indicates that more preventive measures like housing, management, feeding and vaccines are still required.

In the current study, many RWA ear-tags were cut off in the farrowing unit as most of the first time AB treatments were carried out during this period. This suggests, that in order to reduce the number of AB treatment in these RWA herds, interventions are needed in the suckling period. In the present study, BW at 4 weeks of age was identified as the main risk factor in herd A, whereas pigs from litters with less than 19 piglets and barrows were at a higher risk of AB treatment in herd B. Therefore, special attention or interventions should be focused on these subgroups of pigs.

### Bodyweight as a risk factor

Literature is scarce on the effect of BW on disease and AB treatments in pigs. A previous Danish study demonstrated that pigs with a weaning weight below 5.6 kg had an OR of 2.5 of dying in the weaner period compared to pigs weaned above 7.5 kg [12]. Also heavier pigs at weaning are less susceptible to post-weaning diarrhoea [13] and are therefore less likely to be treated with AB in the post-weaning period. This is consistent with the current study, where pigs with a BW below 4.5 kg at 4 weeks of age were at a higher risk of AB treatment in herd A.

Additionally, epidemiological studies have found that pre-weaning diarrhea resulted in a loss of 0.4 kg in a litter at 30 days of age [14], where Johansen et al., [15] reported that being treated for disease or having fore-limb abrasions were negatively associated with ADG. We therefore speculate, whether a low BW has a negative impact on disease frequency in pigs or whether disease affects the growth performance. It has been reported, that IUGR pigs have a slightly different immune response compared to large BW piglets [16] which may cause a higher susceptibility to disease. However, the literature has limited information about the impact of disease on growth performance in pigs. The question therefore remains, whether small pigs have a higher risk of disease and thereby AB treatment, or if pigs are smaller than littermates because they have suffered from a disease?

### Gender as a risk factor

The current study found an OR of 4.0 for barrows associated with AB treatment compared with female pigs at 4 weeks of age. This is not surprising as surgical castration increases the risk of infection for the barrows. Equivalently, previous studies found that castrated male pigs had a 15% higher risk of dying before weaning [12], are more susceptible to mortality during suckling [17] and had an increased mortality in the weaner period [18]. On the other hand, in the current study no effect on gender were observed on AB treatments in herd A. However, the underlying mechanism for this sexual dimorphism in preweaning mortality rates are unknown, but biological differences across sexes may provide an explanation to the present observations.

### Litter size as a risk factor

A previous risk factor study demonstrated a significant negative linear association between the number of total born pigs and mortality in the farrowing unit [12]. In contrasts, this study showed that pigs from litters of less than 19 piglets had a higher risk of AB treatment throughout the study period in herd B. However, due to the low number of pigs treated with AB (5 out of 21), it is impossible to draw conclusions based on these numbers. It does not seem likely, that piglets from litters of less than 19 piglets have a higher risk of AB treatment.

### Other risk factors

Studies have reported that pigs born from a first parity sow have a 85% higher risk of dying compared to pigs from third or higher parity sows [12], and that survivability increased when piglets were nursed by third to fifth parity sows compared to first and second parity sows [19]. In the present study, we found that as a single factor, pigs from first parity sows had a significantly higher risk of AB treatment compared to pigs from multiparous sows in herd B. This may be caused by a lower BiW in pigs from first parity sows as the survivability increases with BiW. However, we recorded no interaction between BiW and AB treatment in the current trial, but the effect of parity may be caused by an indirect effect of the lower BiW of first parity sows [20]. Moreover, piglets from first parity sows are more susceptible to post-weaning diseases due to a reduced adaptive immune responsiveness [21]. This difference did not reflect on the mortality in the study by Miller et al. [21], nor does it seem to have been the case in the current study.

Moving piglets between sows after 2 days of age increases the risk of dying 2.5 times [12] which may be caused by these pigs being latecomers that are not thriving in their litter after equalisation. In the current study, pigs were moved between sows up to four times during suckling, which however did not seem to have a negative impact on the number of AB treatments. Cross-fostering is performed to reduce size variations of nursed litters and fostered pigs therefore has a 40% greater probability of survival compared to pigs raised by their biological mother [19]. On the other hand, pigs from the current study were also moved up to four times during the nursing period which did have an effect as a single factor on AB treatments in herd A. But the significance disappeared in the multivariable analysis and could not be rediscovered in herd B. It can therefore be speculated, whether the impact of transfers in the weaner unit is simply caused by pigs moving in and out of a sick pen, and thereby having an effect on AB treatments.

Unfortunately, the information concerning indications of AB treatments were lacking in herd A, and no further conclusions can be drawn. As for herd B, AB-treatments were given to treat diarrhoea and leg injuries during the first 2 weeks of the trial. This supports previous descriptions for prescribing AB to piglets, as musculoskeletal diseases were reported to be the major reason [22]. This lack of information is clearly a weakness of the study and future research should include not only risk factors for AB treatment but also reasons for AB treatment. Additionally, further research in a larger sample of farms should be conducted in order to fully understand these results, and whether or not these findings can be confirmed in other farms and therefore be extrapolated to other herds.

## Conclusions

Conclusively, in two Danish RWA herds it was recorded that 64 and 68% of liveborn pigs could reach 12 weeks of age without any AB treatments. In herd A small pigs at weaning were at an increased risk of AB treatments, whereas barrows and pigs from litters < 19 pigs were at higher risk of AB treatment in herd B. From this quantitative study it can therefore be suggested that these pigs require increased attention in these two RWA herds. The study confirms that pigs are able to be reared without AB under certain conditions in conventional Danish production systems.

## Supplementary Information

**Additional file 1: Table 1.** Feed composition and chemical analysis of the weaner, grower and finisher pig diets in Herd A.

**Additional file 2: Table 2.** Feed composition and chemical analysis of weaner and grower pig diets in Herd B.

## Data Availability

The datasets used and analyzed during the current study are available from the corresponding author on reasonable request.

## Abbreviations

AB: Antibiotics
ADG: Average daily gain
BiW: Birthweight
BW: Bodyweight
IUGR: Intra-uterine growth restriction
OR: Odds ratio
RWA: Raised without antibiotics
