# Supplementary material for: Performance and risk factors associated with first antibiotic treatment in two herds, raising pigs without antibiotics
Source: Porcine Health Manag. 2021 Feb 17;7:18. doi: 10.1186/s40813-021-00198-y (PMC7888151; doi:10.1186/s40813-021-00198-y)
Supplement: Supplementary file 1 — Additional file 1: Table 1. Feed composition and chemical analysis of the weaner, grower and finisher pig diets in Herd A. [file 40813_2021_198_MOESM1_ESM.docx]

**Additional file 1**

**Table 1** *Feed composition and chemical analysis of the weaner, grower and finisher pig diets in Herd A*

|  | Weaner | | Weaner | Grower | | Grower | | Finisher |
| --- | --- | --- | --- | --- | --- | --- | --- | --- |
| Item | 6-9 kg | | 9-15 kg | 15-30 kg | | 25-35 kg | | 30-100 kg |
| Ingredients (%) |  | |  |  | |  | |  |
| Barley and wheat mix | 64.1 | | 71.6 | 70 | | 76.6 | | 78.5 |
| Soy meal, dehulled, 46% | - | | 8 | 16.6 | | 16.5 | | 17.9 |
| Fermented rapeseed | 6 | | 4 | 3 | | - | | - |
| HP 300 | 11.7 | | 8 | 2.5 | | - | | - |
| Sugar beet pulp | - | | 1.5 | 1.5 | | 2 | | - |
| Danmilk allround | 9 | | - | - | | - | | - |
| Soy oil | 2.6 | | 2.4 | 1.8 | | 0.8 | | 0.5 |
| Premix 1^1^ | 8.6 | | 4.5 | 4.1 | | 4.1 | | 3.1 |
| Premix 2^1^ | 1 | | - | 0.5 | | - | | - |
| Chemical composition (g/kg) | |  | | |  | |  | |
| Dry matter | 878 | | 862 | - | | - | | - |
| Crude protein | 184 | | 175 | 178 | | 158 | | 160 |
| Crude fat | 61 | | 46 | 40 | | 29 | | 26 |
| Ash | 32 | | 40 | 41 | | 37 | | 34 |
| Digestible Lysine | 11.81 | | 11.99 | 11.99 | | 9.80 | | 8.39 |
| Calcium | 7.1 | | 7.9 | 8.2 | | 7.8 | | 7.0 |

^1^Premix from Vilomix A/S, Mørke, Denmark.
