# Supplementary material for: Performance and risk factors associated with first antibiotic treatment in two herds, raising pigs without antibiotics
Source: Porcine Health Manag. 2021 Feb 17;7:18. doi: 10.1186/s40813-021-00198-y (PMC7888151; doi:10.1186/s40813-021-00198-y)
Supplement: Supplementary file 2 — Additional file 2: Table 2. Feed composition and chemical analysis of weaner and grower pig diets in Herd B. [file 40813_2021_198_MOESM2_ESM.docx]

**Additional file 2**

**Table 2** *Feed composition and chemical analysis of weaner and grower pig diets in Herd B*

|  | Weaner | Weaner | Grower |
| --- | --- | --- | --- |
| Item | 5.5-7 kg | 7-10 kg | 10-26 kg |
| Ingredients (%) |  |  |  |
| A-One starter feed^1^ | 100 | - | - |
| Winter barley | - | 20 | 20 |
| Wheat | - | 38.5 | 48.2 |
| Soybean meal, hulled & toasted | - | - | 12 |
| Sow concentrate HP 300 | - | 14 | 9.5 |
| Vegetable oil and fat | - | 2.5 | 3 |
| “Landmix 1” concentrate 8-10 kg^2^ | - | 25 | - |
| “Landmix 3” concentrate 10-26 kg^2^ | - | - | 7.3 |
| Chemical composition (g/kg) |  |  |  |
| Dry matter | - | 89.6 | 88.3 |
| Crude protein | - | 18.4 | 18.5 |
| Crude fat | - | 6.7 | 5.2 |
| Ash | - | 6.1 | 3.6 |
| Lysine | - | 14 | - |
| Digestible Lysine | - | - | 12.1 |
| Calcium | - | 6.6 | 8.9 |

^1^ Wheat, “Sunlustre” soy concentrate, heat-treat wheat, whey powder, lactose powder, soy protein, chicory pulp, dextrose, calcium formiat, vitamins, minerals, soy oil and fatty acids.

^2^Landmix from Vilofoss (Fredericia, Denmark).
